# The Effect of Chronic Deafferentation on Mental Imagery: A Case Study

**DOI:** 10.1371/journal.pone.0042742

**Published:** 2012-08-07

**Authors:** Arjan C. ter Horst, Jonathan Cole, Rob van Lier, Bert Steenbergen

**Affiliations:** 1 Radboud University Nijmegen, Donders Institute for Brain, Cognition and Behaviour, Nijmegen, The Netherlands; 2 Radboud University Nijmegen, Behavioural Science Institute, Nijmegen, The Netherlands; 3 University of Bournemouth, Poole Hospital, Clinical Neurophysiology, Poole, United Kingdom; University of Reading, United Kingdom

## Abstract

Visual- and motor imagery rely primarily on perceptual and motor processes, respectively. In healthy controls, the type of imagery used to solve a task depends on personal preference, task instruction, and task properties. But how does the chronic loss of proprioceptive and tactile sensory inputs from the body periphery influence mental imagery? In a unique case study, we investigated the imagery capabilities of the chronically deafferented patient IW when he was performing a mental rotation task. We found that IW's motor imagery processes were impaired and that visual imagery processes were enhanced compared to controls. These results suggest that kinaesthetic afferent signals from the body periphery play a crucial role in enabling and maintaining central sensorimotor representations and hence the ability to incorporate kinaesthetic information into the imagery processes.

## Introduction

The ability to imagine is regarded as an extraordinary human capacity. Humans are able to mentally manipulate internal representations. This imagery capability is understood as a reconstruction of actual perceptual experience from the past. Two types of imagery that have been studied extensively are visual imagery (VI) and motor imagery (MI). In VI, participants mentally perform visual transformations of an object or scene without a retinal projection of that image [1]. In contrast, MI represents a mental movement of one's own body parts from a first person perspective. MI is thus defined as a dynamic state during which a participant mentally simulates a given action without overt movement [2].

MI, but not VI, has been shown to be subject to postural manipulations [3], [4], [5] and biomechanical constraints [6], [7]. These effects are thought to result from a conflict between the imagined movement and the body's current posture and movement abilities [3], [8]. From studies on amputees it is known that the (partial) loss of the effectors and hence both afferent and efferent kinaesthetic sensations, results in a lack of bodily influences on the MI processes [9], [10], [11]. These studies looked at the necessity of a present effector for the interaction between body representations and imagery processes [10], and the influence of a missing effector on MI processes [11]. There is, however, ambiguity as to the role of the mere kinaesthetic afferent or efferent sensations in the generation of these bodily influences on the MI processes. In a study with a peripherally deafferented patient, Mercier et al. (2008) argued that this conflict mainly arises from online afferent feedback, influencing the MI processes [8]. However, in a recent study, Silva et al. (2011) showed that during transient deafferentation due to local anaesthesia of the arm, MI processes are slower and less accurate overall, but the influence of biomechanical constraints remained. Hence, the loss of kinaesthetic afferents alone is not sufficient to alter the embodied properties of MI processes. Consequently, it is likely that the postural and biomechanical conflicts arise (at least partly) from central processes. We know from previous studies that MI is dependent on centrally constructed body representations [4], [12], which represent the body's current posture and action abilities [13], [14], [15]. Therefore, we examine in the present study how the long-term loss of kinaesthetic afferents influences central imagery processes and specifically, the role of these kinaesthetic afferents on the interaction between the imagined movements and the body's current posture and biomechanical constraints. By doing so, we provide new insight in the selective role of afferent information on (mental) motor processes.

In order to answer this question, we performed two experiments with an individual suffering from a rare case of selective peripheral deafferentation - a condition of selective and complete chronic loss of proprioceptive and tactile afferents due to a sensory neuronopathy [for a more elaborate description of the condition see: 16], [17]. From the literature it is known that the deafferented subjects IW and GL are able to explicitly construct motor representations as both are able to perform accurate movements, although likely with a more visual cognitive supervision than controls [18], [19]. Consequently, and in contrast to Mercier et al. (2008), we used an implicit mental rotation task to study the influence of long-term deafferentation on the implicit use of internal motor representations. We used mental rotation tasks in which MI (Experiment 1 and 2) and VI (Experiment 1) are implicitly induced. The mental rotation task is a well defined task to study imagery [7], [20]. During the task, participants are presented with rotated pictures of corporeal or non-corporeal objects (i.e., hands or letters, respectively). In order to solve mental rotation tasks, participants use visual and motor based strategies. Without explicit task instructions on how to solve the task, the use of non-corporeal objects without accompanying motor representations results in the implicit use of VI [21]. The presentation of corporeal objects results in the implicit use of MI [22]. In order to establish whether MI or VI was used, we manipulated the participants' posture during both experiments and measured the influence of biomechanical constraints on the imagery processes. The influence of biomechanical constraints during the mental rotation can be defined as the difference in performance between hand stimuli rotated toward and away from the body's midsagital plane [6], [7], [23], [24], [25]. Furthermore, in order to be able to ascribe possible effects to the deafferentation and not to handedness, we included both left and right handed age and sex matched controls as IW is strongly left handed. If IW is able to construct a representation of his current body posture and his action abilities, we expected to find postural and biomechanical influences on MI. If, on the other hand, the long-term loss of afferent information prevents IW constructing a postural and biomechanical representation as controls, we expected to find a lack of postural and biomechanical influence on MI. Furthermore, we expected IW to outperform controls on the VI tasks as IW is used to visualizing not only his own movements prior to execution and movement rehearsal [26] but also the movements of others for anticipation in daily life.

## Materials and Methods

### Participants

The study was approved by the ethics committee of the Faculty of Behavioural Sciences from the Radboud University Nijmegen and all participants gave written informed consent prior to the experiment, in accordance with the Helsinki declaration. The tasks in both experiments were performed by the deafferented person IW (age 59 years, male, left-handed), fifteen left-handed controls (mean age 57.1 years, range 51–61 years), denoted as CL, and fifteen right-handed controls (mean age 56.3 years, range 51–65 years), denoted as CR. All controls were neurologically healthy and age and sex matched to IW (z-score IW vs. CL: 0.58 and IW vs. CR: 0.45). Hand preference was assessed according to the Edinburgh Handedness Inventory [27]. Hand preference was found in all participants (laterality quotient: IW, −100; left-handed participants, −54±25.7 mean ± SD and right-handed participants, 90±18.5 mean ± SD). All participants had normal or corrected-to-normal vision.

### Stimuli

**Figure 1:**
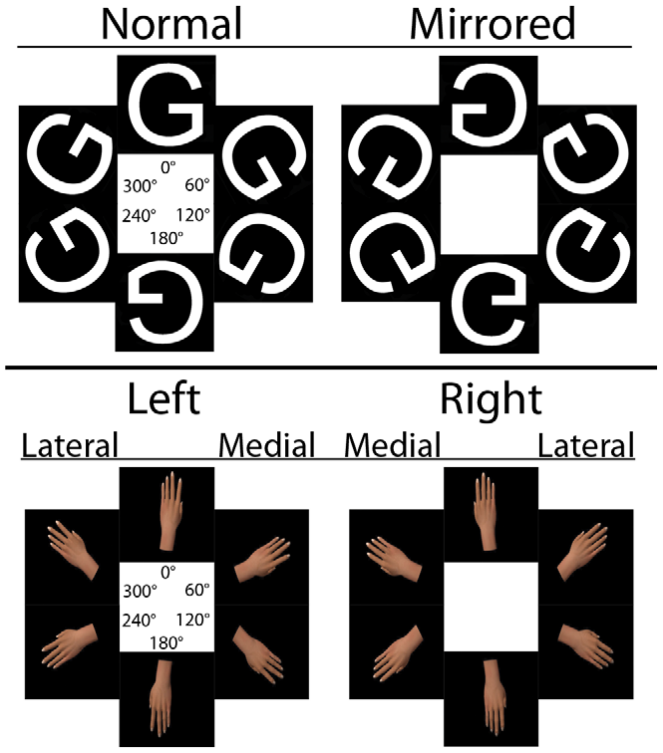
Examples of used stimuli. Examples of stimuli as used in Experiment 1 (letters and hands) and Experiment 2 (hands). Degrees represent the in-plane rotational angle.

As corporeal stimuli we used a custom made 3D hand model designed in a 3D image software package (Autodesk Maya 2009, USA). From this realistic model we constructed all corporeal stimuli that were used in Experiment 1 and 2. The hand-stimuli were shown from both a back- and palm view, see Figure 1. Additionally, as non-corporeal objects, we used typographical character stimuli for Experiment 1 in Times New Roman font, shown in a canonical or mirrored orientation, see Figure 1. The stimuli were displayed on a 19” LCD computer screen, at a distance of approximately 70 cm from the participants' eyes, resulting in a vertical visual angle of approximately 6°. All stimuli were shown in six different angles of in-plane rotation (i.e. 0°, 60°, 120°, 180°, 240° and 300°), resulting in 24 unique hand stimuli and 24 unique letter stimuli.

### Procedure

#### Experiment 1

The participants were placed in front of a computer screen. Stimulus presentation was controlled using custom developed software in Presentation (Neurobehavioral systems, Albany, USA). Prior to the stimulus a fixation cross was presented at the centre of the screen for a random time between 800 ms and 1200 ms. After this, the stimulus was presented and visible until a response was given. A response consisted of the words “left” or “right” for hand stimuli and “normal” or “mirror” for letter stimuli. RTs were automatically recorded by use of a microphone detecting supra-threshold responses. Response accuracy was manually recorded by an experimenter during the experiment. After the response, a black screen was displayed for 800 ms. Stimuli were presented one at a time. Participants were instructed to judge the laterality of the hand-stimuli or the mirrored or canonical presentation of the letter-stimuli as fast and as accurately as possible, without explicit instructions on how to solve the task. Participants were tested in one experimental session consisting of eight blocks. For each stimulus type (i.e. letter and hands) the participants positioned their hands on their lap underneath the table with the palms oriented downward in two blocks. In the other two blocks, participants positioned their hands behind their back with their fingers intertwined [4]. Consequently, the participants performed four blocks with hand-stimuli and four blocks with letter-stimuli. All stimuli were repeated 4 times, resulting in eight blocks of 48 stimuli. The experiment was preceded by a test of 24 stimuli to familiarize the participants with the task. The order of hand position and stimulus type was randomized and counter-balanced per block.

#### Experiment 2

In the second experiment, the participants were presented with hand-stimuli identical to those used in Experiment 1. In contrast to Experiment 1, the participants positioned their hands on their laps with the palms oriented downward or upward [28]. Furthermore, visual feedback was altered during the experiments. During four of the eight blocks, the participants' hands were covered by a black cloth in order to prevent them from seeing their own hands. During the blocks in which visual feedback of the hands was impossible, the hand position of the participant was changed passively by the experimenter. In the case of IW, he then had no knowledge of the position of his hands, something checked verbally. All other parameters were identical to those of Experiment 1.

### Data analysis

#### Experiment 1

Reaction times smaller than 400 ms and larger than 3500 ms were excluded from further analyses (total loss 3.7% of all trials) in correspondence with former studies [20], [29]. Reaction time analyses were then performed on correct responses. Incorrect responses were a ‘left’ response for a ‘right’ hand or a ‘mirrored’ response for a ‘canonically’ oriented letter and vice versa. Analyses on accuracy data were performed on the percentage of correct responses.

The effect of the different conditions in the control participants was assessed using separate mixed design analyses of variance (ANOVA) for testing the influence of postural changes and biomechanical constraints. The rationale for using different ANOVAs is that letter stimuli in general, and 0° and 180° rotated hand stimuli, cannot be denoted as being laterally or medially rotated. Furthermore, this method provides a single numerical measure for the influence of the biomechanical constraints for comparing the biomechanical influences between the control groups and IW. In order to test the postural influence on performance of the controls we used an with the following design: 1 between subject variable Group, with two levels: Control Left (CL) and Control Right (CR); 3 within-subject factors (Type, Posture and Angle), with 2 levels for Type (Letter, Hand), 2 levels for Posture (on lap, behind back) and 4 levels for Angle (0°, 60°, 120° and 180°). The values labelled 60° and 120° are the averaged RTs of 60° and 300°, and 120° and 240° rotated stimuli, respectively. To test for the influence of biomechanical constraints on the performance for hand stimuli we used an ANOVA with 1 between subject variable (Group) with two levels (CL, CR) and 1 within subject variable Direction Of Rotation (DOR) with two levels (Lateral rotations, Medial rotations).

Individual results of IW were analyzed using separate non parametric Friedman's tests for both types of stimuli (i.e. hands and letters) with Angle as factor with 4 levels (0°, 60°, 120° and 180°). Wilcoxon Signed Rank Tests were used as post-hoc tests. The Wilcoxon Signed Rank test was also used to test for postural influences for both hand and letter stimuli separately. The same non-parametric test was used to test for differences between lateral and medial rotations.

To compare the results of IW with those of the CL and CR groups, we used 95% confidence intervals (CI). We calculated difference scores across trials of the postural effects (i.e. hands behind the back>hands on the lap) for both stimulus types for IW, CL and CR. Furthermore, we also calculated difference scores for the DOR-effect for hand stimuli only (i.e., lateral>medial). From these (difference) scores, we calculated the 95% CI based on the t-distribution for CL and CR and determined whether IW's difference scores fell outside the confidence intervals [8].

Accuracy data were analyzed using the same statistical designs as for the RT data. Post hoc analyses were Bonferroni corrected and the alpha-level was set at *p* = 0.05.

#### Experiment 2

For experiment 2, the same exclusion criteria were used as for Experiment 1, resulting in 4.2% loss of trials. In this second experiment we were interested in the effects of the postural manipulations, biomechanical constraints and the modulation of visual feedback in the performance for hand stimuli in the different groups. As in Experiment 1, we used different mixed design ANOVAs. For testing the postural influence and the effect of changing the visual feedback we used a mixed design ANOVA with the following design: 1 between subject variable Group, with two levels: Control Left (CL) and Control Right (CR); 3 within-subject factors (Feedback, Congruency and Angle); with 2 levels for Feedback (Seen, Unseen), 2 levels for Congruency (Congruent, Incongruent) and 4 levels for Angle (0°, 60°, 120° and 180°). To test the influence of biomechanical constraints we used an identical test as in Experiment 1. Individual results of IW were analyzed as in Experiment 1 for the factors Angle, Feedback, Congruency and biomechanical constraints. Identical tests as in Experiment 1 were used to compare the results of IW with those of the left- and right handed controls. Accuracy data were analyzed using the same statistical designs as for the RT data. Post hoc analyses were Bonferroni corrected and alpha-level was set at *p* = 0.05.

## Experiment 1

### Results

**Figure 2:**
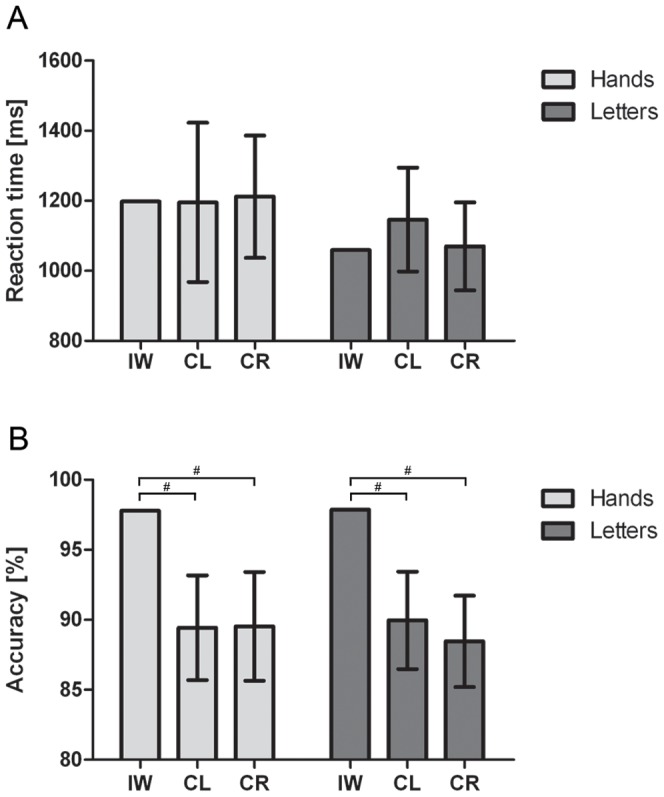
Mean reaction times and accuracy in Experiment 1. Reaction time (A) and accuracy data (B) for IW, CL and CR for Experiment 1. Error bars represent the 95% CI. # denotes that the mean score of IW falls outside the 95% CI of the control group.

**Figure 3:**
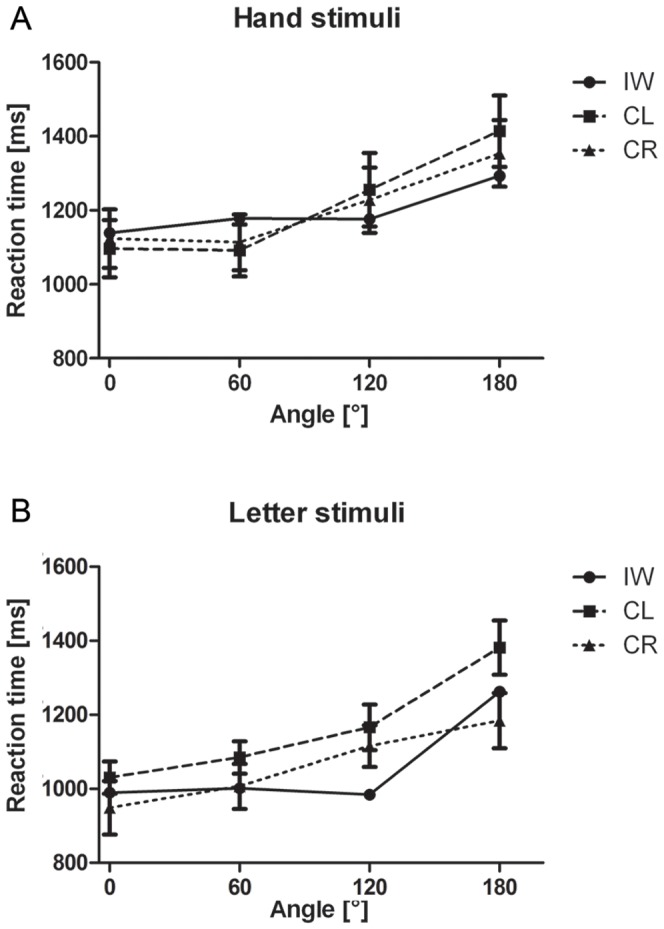
Reaction times as function of rotational angle in Experiment 1. Reaction time data from Experiment 1 as function of angle for hand stimuli (A) and letter stimuli (B). Error bars represent standard error of the mean.

**Figure 4:**
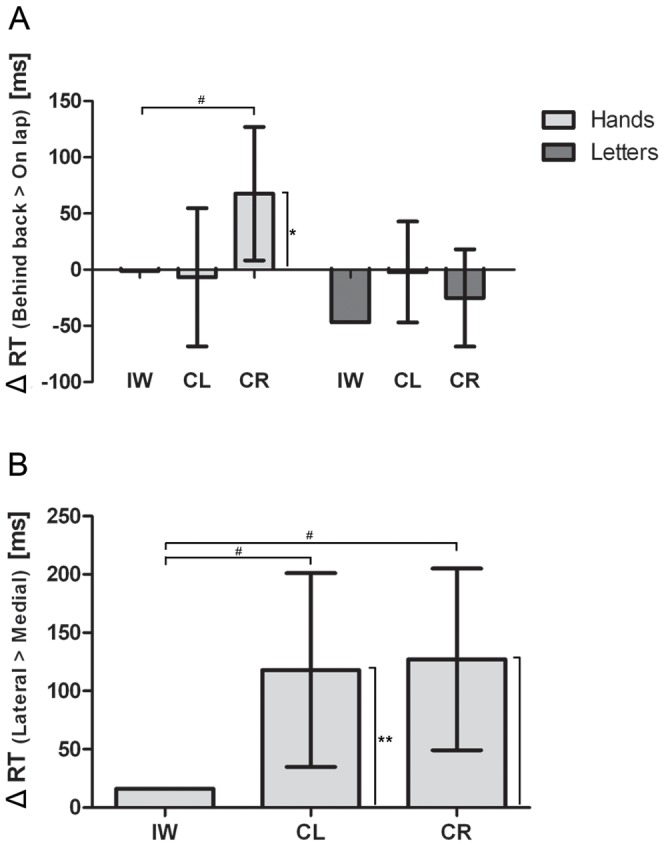
Difference scores for postural and biomechanical influences in Experiment 1. Differences in reaction times between the ‘hands behind the back’ and ‘hands on lap’ postural conditions (A) and between lateral and medial rotations for IW, CL and CR (B). Error bars represent 95% CI. * denotes significance at the *p*<0.05 level, ** denotes significant at the *p*<0.01 level and # denotes that the mean score of IW falls outside the 95% CI of the control group.

For the correct responses, the overall RT of IW did not differ from the control groups, see Figure 2a. The mixed design ANOVA revealed a significant main effect of Angle (F(3,84) = 90.270; *p*<0.001; η^2^ = 0.763). Despite a significant interaction between Angle and Group (F(3,84) = 3.214; *p*<0.05; η^2^ = 0.103), both groups showed a significant simple effect of Angle (CL: F(3,42) = 64.451; *p*<0.001; η^2^ = 0.822 and CR: F(3, 24) = 30.050; *p*<0.001; η^2^ = 0.682), see Figure 3. Furthermore, we obtained a significant interaction of Type by Posture (F(1,14) = 6.212; *p*<0.05; η^2^ = 0.307). Further simple effect analyses revealed no significant postural influences for neither of the stimulus types (all *p*>0.07). Crucially, we obtained a significant three-way interaction of Type by Posture by Group (F(1,28) = 4.412; *p*<0.05; η^2^ = 0.136). Further analyses for the CL group revealed no significant effect of posture or interaction of Type by Posture (all *p*>0.75). For the CR group, however, we obtained a significant interaction of Type by Posture (F(1,14) = 6.212; *p*<0.05; η^2^ = 0.307), which resulted in a significant simple effect of Posture (F(1,14) = 6.643; *p*<0.05; η^2^ = 0.322) only for the hand stimuli and not for the letter stimuli (*p*>0.55), see Figure 4a. For IW we only obtained a significant effect of Angle for both the Hand stimuli (χ^2^(3) = 10.275, *p*<.02) and Letter stimuli (χ^2^(3) = 26.625, *p*<.001), see Figure 3. The postural influence found for the hand stimuli in the CR group differed significantly from the postural influence obtained for IW, see Figure 4a. The mixed design ANOVA on the biomechanical constraints revealed a significant mean effect of the DOR (F(1,28) = 26.288; *p*<0.001; η^2^ = 0.484). This effect was not modulated by Group (*p*>0.95), see Figure 4b. For IW, the influence of the biomechanical constraints was not significant (*p*>0.54). The influence of the biomechanical constraints for IW differed significantly from that of both control groups, see Figure 4b.

For the accuracy data we obtained a significant interaction of Angle by Group (F(3,84) = 6.050; *p*<0.005; η^2^ = 0.178). Further analyses revealed a decrease in accuracy as function of the angular rotation for CL (F(3,42) = 13.791; *p*<0.001; η^2^ = 0.496) and CR (F(3,42) = 32.416; *p*<0.001; η^2^ = 0.698). No further effects were found significant. In the comparison of the accuracy between the control groups and IW we found that IW was significantly more accurate than both control groups, see Figure 3b.

### Discussion

In Experiment 1, we studied the influence of deafferentation on imagery capabilities. We expected IW's MI to be impaired and his VI to be enhanced compared to controls. We found that controls showed the typical RT and accuracy profiles for mental rotation tasks for the letter and hand stimuli [6], [7], [20], [22]. Hence, we can conclude, that the controls did use a mental rotation strategy to solve the task. For IW, though we also found significant Angle effects for both stimulus types, we only found a gradual increase in RTs as a function of rotational angle for the hand stimuli. The RT profile for the letter stimuli showed nearly equal RTs from 0° to 120°, all differing significantly from 180°, see Figure 3. Therefore, IW seemingly does not use a mental rotation strategy. However, during analysis if IW's introspection, he reported that he mentally “*placed the letter on an imaginary disc in order to rotate it upward*”. This implies that IW did use a mental rotation strategy, albeit a modified one.

The postural manipulations did not influence IW's performance during the mental rotation of letters and hands. For the letter stimuli, the difference scores for the postural manipulations between IW and the controls did not differ, see Figure 4a. This finding is intuitive and agrees with the literature; letter stimuli are non-corporeal objects and hence do not implicitly induce egocentric processing [20]. For the hand stimuli, the lack of postural influence for IW differed significantly from the CR group, but not from the CL group, see Figure 4a. The lack of postural influence for IW is in correspondence with Mercier et al. (2008), who showed that kinaesthetic afferents are an important factor in the modulation of the imagery processes. However, Mercier et al. (2008) also showed that the ability to see one's own hand during the task can result in the construction of a representation of the current posture from available visual feedback, thereby interfering with the imagined movement. In Experiment 1, the visibility of the participants' hands was confined with the postural manipulation. That is, during the placing of the hands in the correct position in the “hands on lap” and the “hands behind the back” conditions, the participants' hands were visible and invisible, respectively. Therefore, in Experiment 2, the participants' posture and visibility of the hands were manipulated separately during a mental rotation task of hands. If IW is able to construct a visual representation of his hands' current position and incorporates this representation into his planned movement, we would expect an influence of the hand posture on the performance only when IW is able to observe his own hands. In contrast, when IW does not construct a visual representation of his hands' position, IW would not show any postural influence, irrespective of the ability to see his own hands.

## Experiment 2

### Results

**Figure 5:**
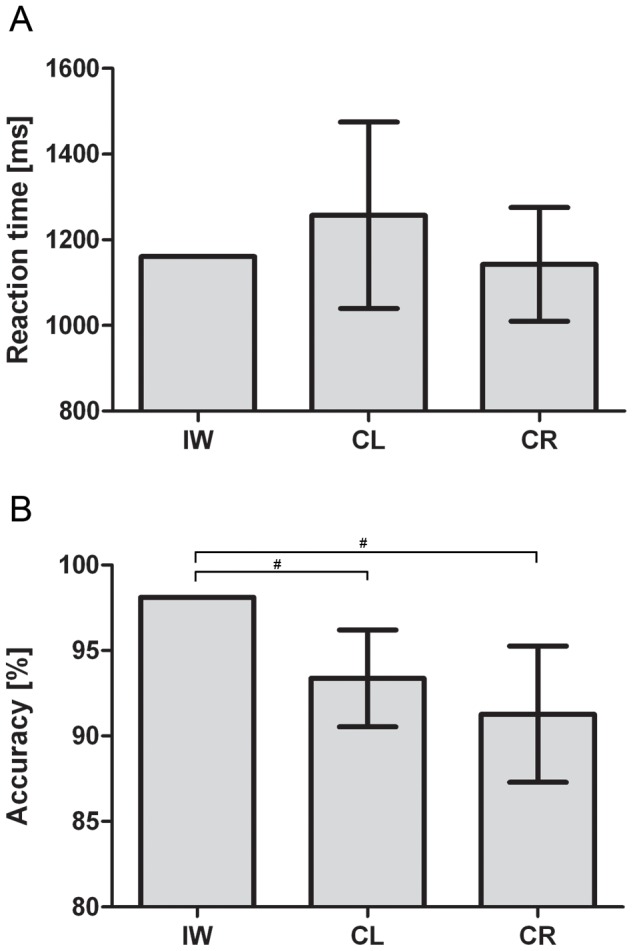
Mean reaction times and accuracy in Experiment 2. Reaction time (A) and accuracy data (B) for IW, CL and CR for Experiment 1. Error bars represent the 95% CI. # denotes that the mean score of IW falls outside the 95% CI of the control group.

**Figure 6:**
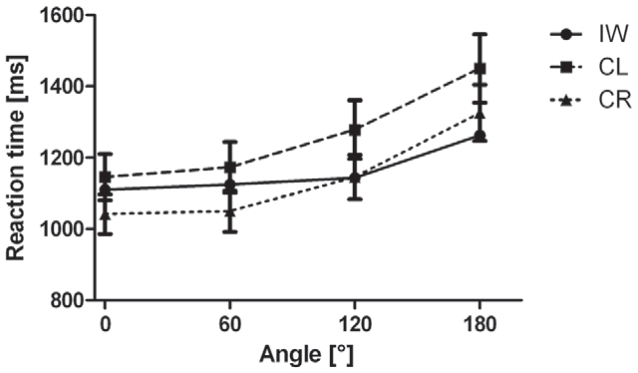
Reaction times as function of rotational angle in Experiment 2. Reaction time data from Experiment 1 as function of angle for hand stimuli. Error bars represent standard error of the mean.

**Figure 7:**
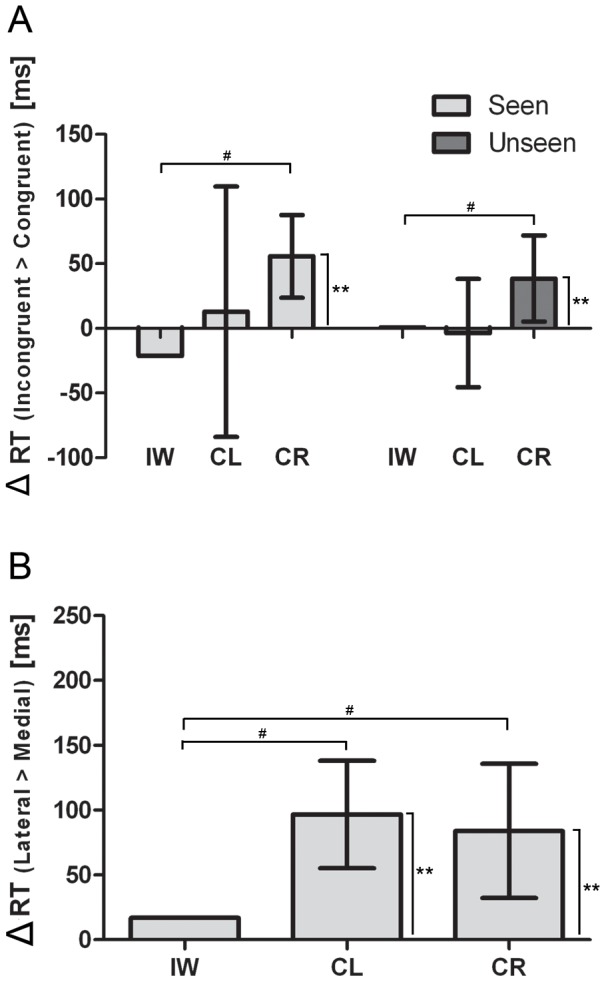
Difference scores for postural and biomechanical influences in Experiment 2. Differences in reaction times between the ‘Incongruent’ and ‘Congruent’ postural conditions (A) and between lateral and medial rotations for IW, CL and CR (B). Error bars represent 95% CI. * denotes significance at the *p*<0.05 level, ** denotes significant at the *p*<0.01 level and # denotes that the mean score of IW falls outside the 95% CI of the control group.

In line with Experiment 1, we found for the correct responses that the overall RT of IW did not differ from the control groups, see Figure 5a. The mixed design ANOVA revealed a significant main effect of Angle (F(3,84) = 74.131; *p*<0.001; η^2^ = 0.726) and Congruency (F(1,28) = 4.621; *p*<0.05; η^2^ = 0.142), see Figure 6. Despite a significant interaction of Congruency by Angle (F(3,84) = 6.695; *p*<0.002; η^2^ = 0.193) we obtained significant simple effects of Angle for the Congruent (F(3,87) = 84.478; *p*<0.001; η^2^ = 0.744) and Incongruent conditions (F(3,87) = 40.696; *p*<0.001; η^2^ = 0.584). Crucially, we obtained a significant two-way interaction of Congruency by Group (F(1,28) = 6.040; *p*<0.02; η^2^ = 0.177). Further simple effect analyses for the CL group revealed no significant effect of Congruency (*p*>0.82). For the CR group, however, we obtained a significant simple effect of Congruency (F(1,14) = 10.048; *p*<0.01; η^2^ = 0.418), see Figure 7a. For IW we only obtained a significant effect of Angle (χ^2^(3) = 16.350, *p*<.001) and no influence of the postural manipulation irrespective of the feedback manipulation (all *p*<0.09), see figures 6 and 7a, respectively. The postural influence found for the CR group differed significantly from the postural influence obtained for IW, see Figure 7a. The mixed design ANOVA on the biomechanical constraints revealed a significant mean effect of the DOR (F(1,28) = 35.138; *p*<0.001; η^2^ = 0.557). This effect was not modulated by Group (*p*>0.95), see Figure 7b. For IW, the influence of the biomechanical constraints was not significant (*p*>0.63), see Figure 7b. As in Experiment 1, the influence of the biomechanical constraints for IW differed significantly from that of both control groups, see Figure 7b.

For the accuracy data we obtained a significant effect of Angle (F(3,84) = 26.492; *p*<0.001; η^2^ = 0.486). No further effects were found significant. In the comparison of the accuracy between the control groups and IW we found that IW was significantly more accurate than both control groups, see Figure 5a.

### Discussion

In this experiment, we were interested in the influence of visual feedback on the effect of postural manipulations for IW. In correspondence with the findings of Experiment 1, we found that IW was significantly more accurate than the CR and CL group and that the significant DOR effects for both control groups differed significantly from IW, see Figure 7b. For the postural manipulations we found that IW's lack of postural influence differed significantly from the significant posture effect for the CR group, irrespective of the visual feedback. Consequently, these findings confirm that kinaesthetic feedback plays an important role in the emergence of a postural conflict irrespective of the visual feedback on the effectors involved. Furthermore, the ability to visually observe one's own hand apparently does not evoke the use of a visually constructed representation of the hand by IW in a mental rotation task of hands.

## General Discussion

In the current case study we investigated the influence of deafferentation on imagery capabilities. We specifically looked at the role of kinaesthetic afferents and how the imagery processes are affected due to long term loss of afferent input. We expected IW's MI to be impaired and that his VI might be enhanced compared to controls.

In both experiments, we found that IW's performance was not influenced by postural manipulations, irrespective of the ability to see his own hands. For the controls, we found that the CR group was influenced by the postural manipulations but not the CL group. This latter was expected and is considered to be related to differing internal representations of the hands between left- and right handed people [30], [31]. Because both the CL group and IW show a lack of postural influence, one might argue that this is attributable to handedness alone, because IW is left handed. However, the influence of biomechanical constraints (as reflected in the DOR-effect) differed significantly between IW and both control groups in both experiments, see Figure 4 and 7. Consequently, the lack of embodied influences for IW cannot be solely attributed to handedness alone and is therefore likely to result from the lack of kinaesthetic afferents for IW.

Collectively, our results show that the long-term loss of kinaesthetic afferents results in an inability to implicitly incorporate kinaesthetic information into one's centrally generated body representations. Clearly, due to deafferentation there is no direct kinaesthetic feedback to incorporate into a body representation. Interestingly it is likely that in addition, the long-term loss of kinaesthetic afferents also results in an inability to recall these sensations from memory in order to incorporate them into the body representation. Memory has been shown to play a role during MI [32]. The role of memory in MI processes is also evident from the sustained influence of biomechanical constraints during transient anaesthesia of the arm in a MI task [33]. Furthermore, it has been shown that even during transient peripheral deafferentation acute plastic changes occur in the brain [34], [35], also leading to alterations in the central representation of the body in the brain [36]. Additionally, it has also been shown that a lack of experience in the sensation of a certain movement results in an inability to imagine that movement [37]. The emergence of postural and biomechanical conflicts from centrally generated conflicts between body representations and imagined movements is in line with recent experimental results [38]. Furthermore, it is also in line with the emulation theory [39], which states that body representations (represented in the ‘emulator’) play a crucial role in MI processes and are constructed from former experience of afferent sensations [40]. Consequently, by providing experimental evidence, our results give further rise to the notion that MI is a centrally generated, offline process [41].

This does not imply that IW is unable to construct central representations of the body's current position. IW is able to perform accurate movements by visualizing his movement from a first person perspective prior to the overt movement [16], [42]. His ability to construct a representation of the body with only visual information is in line with the multimodal nature of body representations [13], [40]. Consequently, IW is able to construct a ‘sensorimotor’ representation. However, this representation is phenomenologically different from the motor representations of controls [18], [19] and is likely to be primarily based on visual perception.

As MI consists of a mental transformation of visual and kinaesthetic percept's [20], it is important to show that the observed lack of postural and biomechanical influences for IW results from affected central motor processes and not an affected ability to create and transform mental images. The results of the letter task show that IW is perfectly able to perform mental transformations of mental images. This is evidenced by the similar performance in reaction times and enhanced accuracy compared to controls. Therefore, it is likely that the observed lack of postural and biomechanical influences for IW results from affected central motor processes due to the chronic deafferentation.

In addition, as IW controls movement with mental attention and close visual supervision, it is also likely that he developed visual mental images of his own body as well. Consequently, his mental transformation skills of visual images is not likely to be limited to letters only. Indeed, we found that IW outperformed both control groups on the accuracy level. with remarkably high accuracy levels between 95% and 100% for all angles. Because IW is unable to mentally simulate the kinaesthetic consequences of a movement, it is likely that IW used VI to solve the mental rotation tasks, irrespective of the used types of stimuli.

Our results show that these VI abilities of IW are enhanced with respect to controls. This high accuracy may be a result of two processes. First, he uses visual imagery of movement in everyday life and used it in his rehabilitation and secondly he seems to have a high level of focused attention. It has already been shown that participants with higher focused attention scores have an increased performance in mental rotation tasks than participants with a lower focused attention score [43]. In daily life, IW has to continuously update his visual percept of the world and translate that knowledge into a motor plan. Diminished attention or errors in the mental transformations are likely to result in improper movements and hence the risk of falling or not being able to grasp an object, for example. He is quite clear that he mentally rehearses movements beforehand and uses visual imagery frequently and widely to maintain performance.

In conclusion, this study provides new insights in the debate on the influence of afferent information in MI processes. In contrast to former studies on the influence of (congenital) amputations on MI processes, we selectively looked at the influence of afferent information on these processes. We found that kinaesthetic afferents play an important role in the conflict between imagined movement and the body's current posture. The body's current posture and biomechanical constraints are likely to be incorporated in a structural body description and processed centrally during imagery. The long term loss of kinaesthetic afferents results in the loss of central kinaesthetic representations and hence impaired motor imagery. In order to compensate for this deficit, IW uses a visual construct of his body, together with online visual supervision to plan and control imagined motor acts. This extraordinary faculty developed over years for planning and indeed predicting movement is likely to explain IW's enhanced visual imagery capabilities [25].
